# Implementing a workflow-integrated motivational interviewing training program for psychiatry trainees on an inpatient consultation-liaison rotation: lessons learned

**DOI:** 10.3389/fpsyt.2023.1184053

**Published:** 2023-05-18

**Authors:** James P. Loveless, Jordan H. Rosen, Joanna S. Yost

**Affiliations:** ^1^Department of Psychology, Middle Tennessee State University, Murfreesboro, TN, United States; ^2^Brigham and Women’s Hospital, Boston, MA, United States; ^3^Department of Psychiatry and Neurobehavioral Sciences, University of Virginia, Charlottesville, VA, United States

**Keywords:** consultation-liaison psychiatry, motivational interviewing, training, psychosomatics, clinical health psychology, inpatient care

## Abstract

**Background:**

Effective consultation-liaison psychiatry (CLP) is proactive, collaborative, and requires providers to have proficiency with therapeutic skills beyond nosology and medication management. Motivational interviewing (MI) is an evidenced-based intervention that should be considered essential for CLP trainees to learn. Given that the demands of training and patient care are already experienced as stressful for many psychiatry trainees, the authors endeavored to create a MI training program that was integrated into trainees’ normal CLP workflow.

**Method:**

Twenty-two trainees on an inpatient CLP rotation participated in a six-week MI training program that was incorporated into their regular workflow. The program included didactic sessions with role-playing, as well as on-demand between-session coaching via an expert in MI. Trainee participation and perceptions of MI were measured via a questionnaire that was administered prior to each training session.

**Results:**

Trainee participation in the didactic sessions was inconsistent. Questionnaire data revealed positive baseline perceptions of motivational interviewing and its usefulness in inpatient medical settings. Additionally, as trainees participated in the program, perceived knowledge of motivational interviewing as well as awareness of motivational issues among their patients increased. Finally, participation in program was not perceived as disruptive to daily workflow for the participants.

**Discussion:**

This the first documented attempt at implementing a MI training program for CLP trainees that was integrated into their regular workflow. Preliminary data identified some encouraging trends, but also unexpected challenges. These lessons could inform how these types of training programs are implemented moving forward.

## Introduction

An estimated third to half of medical inpatients have psychiatric comorbidities, with the most common being unipolar mood disorders, neurocognitive disorders, substance use disorders, psychosomatic disorders, and anxiety disorders (1, 2). There is evidence to suggest that the presence of these and other psychiatric conditions adversely impact care, leading to poorer outcomes, increased lengths of stay, and higher treatment-related costs (3–6). Inpatient consultation-liaison psychiatry (CLP) services are often called on to provide care for these patients.

During the past 20 years, models of CLP have evolved to better meet the demands of contemporary inpatient medicine. Traditional models of CLP follow a reactive approach wherein intervention for identified acute mental health needs occur only after a referral is made from a member of the primary medical team. While these models offer some additional care to patients and support to primary teams, they have also been criticized as siloed, inefficient, and ineffective (1). Newer models of CLP, on the other hand, emphasize a more proactive approach to care to so that mental health needs can be identified and ameliorated as quickly as possible. While several different proactive CLP models have been published in the literature, they all seem to emphasize greater collaboration between the CLP team and primary medical teams, increased integration of allied professionals within the CLP team, and early screening and intervention among patients with appreciable psychiatric symptomology (1, 7). Available data seem to indicate that these novel models of CLP are more effective than traditional approaches at reducing length of stay and breaking down many of previously identified barriers to CLP utilization (1, 8–11).

The evolution of CLP has necessitated changes in how residents and medical students are prepared for this kind of work. Contemporary training standards for CLP emphasize the importance of establishing competency across several domains including knowledge, skills, and attitudes (12). Within the skills domain, training programs are expected to train residents and medical students in effective interpersonal skills so that proficiency with rapport building, clinical interviewing, and fostering of productive collaborations among allied health providers (both within and without the CLP team) might be developed. Additionally, training programs are expected to help trainees develop basic competence in broad range of evidence-based psychotherapeutic intervention strategies appropriate to the settings in which they are working (12).

Motivational interviewing (MI) is a formal psychotherapeutic intervention that uses effective interpersonal skills to foster a strong therapeutic alliance, and then leverages that therapeutic alliance in such a way to enhance patient motivation for healthful change (13, 14). It offers a framework by which providers and patients can collaboratively explore and resolve ambivalence to health behavior change, identify and problem-solve barriers to health behavior change, and monitor health behavior change success. MI has been demonstrated to be an effective intervention for many of the behaviorally mediated issues that CLP services treat including substance abuse and non-adherence to treatment, and it has increasingly been utilized in a variety of healthcare setting by physicians and staff alike to improve patient outcomes (14, 15). MI should be considered an essential intervention for CLP trainees to learn as it dovetails nicely with CLP training standards pertaining to interpersonal skills and psychotherapeutic intervention (12, 13).

There is significant heterogeneity in how healthcare providers are trained in MI. The most effective training programs seem to include didactic instruction, experiential learning exercises, and some form of follow-up coaching (16, 17). Despite these common components, there is no standardization with respect to training structure and duration (18). Conventional wisdom favors an approach to training that is highly structured that occurs over the course of hours or days, with follow-up coaching occurring at some regular interval thereafter.

With respect to CLP trainees, such an approach to training could be burdensome given the already high demands upon their time and resources. Indeed, the psychiatry training literature provides ample evidence that psychiatry trainees suffer stress, burnout, depression at higher rates than their peers in other medical specialties (19), and that organizational factors and training demands have been identified as risk factors that likely contribute to the development of those negative training outcomes (19–22). Training experiences that are more integrated into trainees’ regular routines are less likely to be disruptive to trainee workflow and are therefore less likely to contribute to the development of burnout (23, 24).

Given the apparent value of MI as essential tool for the CLP professional but recognizing the need for MI training to be minimally burdensome to CLP trainees, we devised a MI training program that was incorporated into trainees’ existing training routine. What follows is a description of the training program, a review of some preliminary data gathered from the participating CLP trainees, and a discussion of lessons learned.

## Method

The University of Virginia Health System CLP service is a hybrid model that includes both traditional consultation services and proactive services for patients who have exhibited a behavioral emergency during their hospitalization. On any given day, the service is typically comprised of a CLP attending psychiatrist, nurse liaison, three to five psychiatry residents, and three medical students. The CLP service provides consultation for patients on the medical inpatient units in a 608-bed academic medical facility with a Level one Trauma Center.

The trainees who participated in this pilot program included 11 psychiatry residents, 11 medical students, and one trainee who declined to indicate their level of training. All were all on rotation with our CLP service. The psychiatry residents included five PGY-1 s, four PGY-2 s, and two PGY-4 s, while all medical students were completing their psychiatry clerkships. This program was determined by the University of Virginia Health System Institutional Review Board to be a quality improvement project, so informed consent was not obtained from the trainees for their participation; nevertheless, the program and its purpose were explained to the trainees, and all provided their assent to participate.

The MI training was designed to take place within the context of trainees’ regular training schedule and was conducted by an expert consultation-liaison (CL) psychologist, a postdoctoral fellow in clinical psychology, and a CLP attending psychiatrist. It consisted of six, weekly sessions that regularly occurred during CLP rounds. The first training session was an orientation to MI, whereas subsequent sessions focused on skills acquisition. Skills acquisition sessions included a 10-min didactic presentation with a supervised 15-min roleplay exercise in which all available trainees participated. Following each of these training sessions, trainees were encouraged to practice learned skills with patients during CLP rounds throughout the remainder of the week under the guidance of the CL psychologist.

The content of the training program was developed from the work of Miller and Rollnick (25). The first session consisted of an overview of MI, including its theoretical underpinnings, objectives, and the four processes by which those objectives are achieved. The second session focused on practicing effective interviewing and attending skills, whereas the third session oriented trainees to purposeful use of those skills to facilitate engagement with their patients. The fourth session taught trainees how to focus the clinical interview and evoke change talk, and the fifth session explored shared decision making and collaborative planning for health behavior change. The sixth and final session was dedicated to the integration of these different skills.

Trainee participation and perceptions of MI were measured via a questionnaire that we developed with the input of other expert colleagues working within our health center. It consisted of 11 items, with eight items pertaining to trainee perceptions of MI. For these items, trainees were asked to indicate how much they agreed to each using a five-point Likert-scale (1 = “Agree”; 5 = “Disagree”). A list of the questionnaire’s items can be found in Table 1. Trainees were administered the questionnaire prior to each training session.

**Table 1:** Motivational interviewing (MI) pre-training questionnaire items.

Participation tracking
What is today’s date?
How many MI training sessions have you previously completed?
What year of training are you currently?
Perceptions of MI
The MI training will negatively impact my workflow for the day.
I am likely to use MI interventions on the psychiatry CL service.
I have adequate knowledge of MI interventions.
MI interventions are useful in the medical inpatient setting.
I believe that a patient’s own level of motivation for change is important.
Lack of motivation for change is a significant problem with the patients I see.
My patients’ lack of motivation for change is a significant source of frustration in my work.
I use MI interventions regularly on the psychiatry CL service.

## Results

After one full round of training, trainee participation data revealed that no trainee was able to complete all six training sessions. All trainees attended at least one session; however, only 12 trainees (52%) attended at least two sessions, 7 trainees (30%) attended at least three sessions, 3 trainees (13%) attended at least four sessions, and 1 trainee (4%) attended five sessions.

Pre-training baseline perceptions of MI were largely positive. Trainees indicated that they were likely to use MI on the CLP service (*M _Q2 baseline_* = 1.74, *SD _Q2 baseline_* = 0.69). Furthermore, they endorsed the beliefs that MI interventions are useful in the inpatient medical setting (*M _Q4 baseline_* = 1.56, *SD _Q4 baseline_* = 0.59), and that a patient’s own level of motivation for change is important (*M _Q5 baseline_* = 1.22, *SD _Q5 baseline_* = 0.42). They also endorsed the beliefs that patient motivational issues are often barriers to care (*M _Q6 baseline_* = 2.04, *SD _Q6 baseline_* = 0.64) and that poor patient motivation is a significant source of work frustration (*M _Q7 baseline_* = 2.46, *SD _Q7 baseline_* = 0.89). Trainees indicated a perceived lack of knowledge about MI interventions (*M _Q3 baseline_* = 3.79, *SD _Q3 baseline_* = 1.01), and provided a neutral response as to whether they regularly use MI interventions while providing CLP services (*M _Q8 baseline_* = 3.79, *SD _Q8 baseline_* = 1.01). Finally, they endorsed the conviction that participation in the MI training would not be disruptive to their daily workflow (*M _Q1 baseline_* = 3.78, *SD _Q1 baseline_* = 1.04).

**Figure 1:**
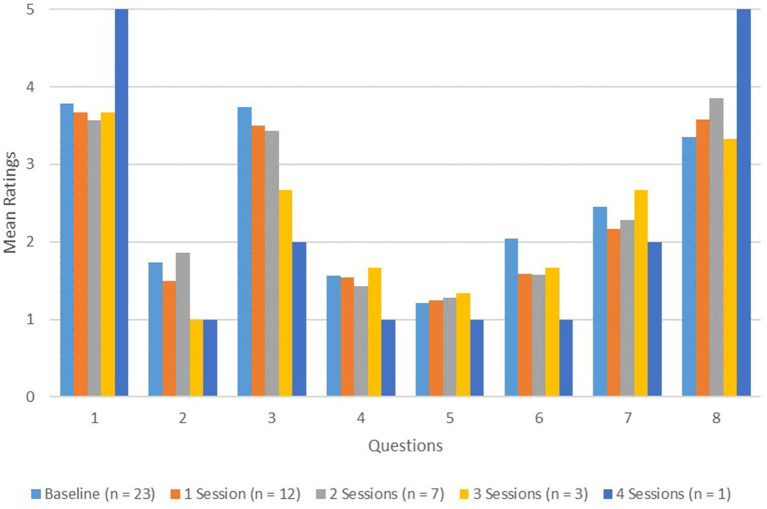
Changes in mean agreement ratings across number of sessions attended.

Within-subjects means testing was not possible with this data as questionnaires were anonymously completed each training session. Nevertheless, certain changes in trainee perceptions were observed as more MI trainings were attended (these can be seen in Figure 1). For example, as trainees attended more training sessions, their perceived knowledge of MI interventions seemed to increase. Likewise, there seemed to be an increased recognition that patient motivation issues are often barriers to care as well as an increased recognition that patient motivation issues caused the trainees frustration at work. Other perceptions did not seem to reliably change in a particular direction. Notably, trainees continued to endorse the belief that MI was useful in inpatient medical settings, as well as the belief that a patient’s own motivation for change is important. They also continued to indicate that they would likely use MI on the CLP service; however, the endorsed regular use of MI while on service also did not seem to change. Finally, trainees’ perception of the disruptiveness of the MI training sessions to their daily workflow also seemed to remain unchanged.

## Discussion

The present paper describes our efforts at creating a MI training program that was minimally disruptive to workflow for psychiatric trainees on rotation with our health center’s inpatient CLP service. To our knowledge, this is the first documented attempt at implementing such a training for trainees while on their CLP rotation. A review of the preliminary data collected from the participants identified some encouraging trends, but also unexpected challenges associated with this type of training approach. Collectively, these lessons could inform how these types of training programs are implemented moving forward.

Participation in this training program seemed to be associated with several encouraging trends in trainee responses. First, trainees perceived knowledge of MI seemed to increase over the course of the program. Second, trainees seemed to gain an increased appreciation of patient motivational issues as notable barriers to care and sources of frustration in their work. Additionally, beliefs about the usefulness of MI in the inpatient medical setting and the importance of patient motivation for change seemed to be maintained throughout the program. Finally, trainees did not perceive the integration of this training into their regular workday as disruptive to their workflow. Taken together, these findings indicate that trainees seemed to benefit from their participation without the training program being perceived as overly burdensome.

Despite the successes of our training program, there were a few unexpected challenges that became apparent during its implementation. For example, the low didactic session completion rate among trainees was identified as a major shortcoming. None of the trainees on the CLP service completed the entire training program, with the majority completing only two sessions. The main reason for this low completion rate likely involved a failure on our part to appreciate the flexibility of scheduling required to meet trainees’ needs. The MI training sessions occurred at a fixed interval which did not account for trainees’ intra-rotation schedules or the occurrence of unexpected events which might interfere with attendance. Future attempts at this type of training program should allow for greater access to training sessions. This could be accomplished through increasing the frequency of weekly training offerings, allowing for a continuous model of training where sessions are cyclical, or the creation and availability of on-demand training resources that could be accessible if a training session is missed.

Yet another apparent shortcoming of our training program was that despite a sustained positive perception of MI and a perceived increase in knowledge of MI, trainees did not seem to endorse an increased use of MI while on rotation. While we could only speculate as to the reasons for this, a review of the MI training literature reveals multiple plausible explanations. For example, trainees might be overestimating their perceived knowledge of MI and their self-reported use is more of a reflection on how much they understand (17). Relatedly, they may fail to see how MI might be applied to their cases or have difficulty transitioning to the role of a collaborator in clinical interactions (26). Finally, trainees might be hesitant to use a set of skills of which they are knowledgeable, but with which are inexperienced (17). In either case, greater access to MI coaching and supervised practice in-between training sessions would likely be helpful at increasing trainee knowledge and comfort with using MI in their CLP work (27–29).

Apart from those already discussed, the MI training literature highlights additional aspects of our training approach which could be modified to improve its quality. For example, the use of self-report surveys in tracking changes in MI proficiency have been criticized in the literature as respondents’ self-reported changes in these measures are only modestly associated with actual changes in clinical behavior (18). Alternative or additional approaches to tracking trainee growth often involve the administration of trainer-rated performance metrics which either involve hypothetical, simulated, or trainee-recorded clinical encounters (30–33). These were considered for use in this project, but we ultimately decided against them for two main reasons. First, these performance metrics were designed to be used in research or in outpatient treatment settings. The practical demands associated with simulating or recording actual patient encounters on acute care inpatient medical units prevented us from seeing how these would be easily utilizable in the current project. Second, the main goal of the project was to develop a MI training experience that was minimally burdensome trainees, and we were concerned that adding additional formal assessment to the experience would be antithetical to that goal. Our choices notwithstanding, a more objective method of tracking trainee learning in this setting is indicated. Future research or quality improvement work in this area should focus on how this can be accomplished.

In sum, we attempted to create a minimally disruptive MI training for psychiatry trainees on rotation with our health system CLP service. Given the behavioral and mental health needs of contemporary medical inpatients, the demand for effective CLP will likely remain high for the foreseeable future. Good training in this area need not be unduly stressful or onerous for trainees. While there are other observations and lesson that could be drawn from the data presented here, we hope that this paper can serve as a starting point for further innovation in how psychiatrists are trained with respect to evidence-based psychotherapeutic interventions that are essential to effective CLP.

## Data availability statement

The raw data supporting the conclusions of this article will be made available by the authors, without undue reservation.

## Ethics statement

The studies involving human participants were reviewed and approved by University of Virginia Institutional Review Board for Health Sciences Research. Written informed consent for participation was not required for this study in accordance with the national legislation and the institutional requirements.

## Author contributions

JL, JR, and JY contributed to the planning and execution of the project. JL managed the data and prepared the manuscript. JR and JY assisted with editing after the manuscript was prepared. All authors contributed to the article and approved the submitted version.

## Conflict of interest

The authors declare that the research was conducted in the absence of any commercial or financial relationships that could be construed as a potential conflict of interest.

## Publisher’s note

All claims expressed in this article are solely those of the authors and do not necessarily represent those of their affiliated organizations, or those of the publisher, the editors and the reviewers. Any product that may be evaluated in this article, or claim that may be made by its manufacturer, is not guaranteed or endorsed by the publisher.
